# Longitudinal comparison of *Streptococcus mutans*-induced aggravation of non-alcoholic steatohepatitis in mice

**DOI:** 10.1080/20002297.2018.1428005

**Published:** 2018-01-22

**Authors:** Shuhei Naka, Kaoruko Wato, Rina Hatakeyama, Rena Okawa, Ryota Nomura, Kazuhiko Nakano

**Affiliations:** ^a^ Department of Pediatric Dentistry, Osaka University Graduate School of Dentistry, Suita, Japan

**Keywords:** *Streptococcus mutans*, non-alcoholic steatohepatitis, longitudinal comparison, mouse model, two-hit theory

## Abstract

**Background**: We previously reported that intravenous administration of *Streptococcus mutans* strain TW871 caused typical non-alcoholic steatohepatitis (NASH)-like findings in a high-fat diet (HFD) mouse model at 16 weeks after initiating the experiment.

**Objective**: The purpose of the present study was to analyse mice administered *S. mutans* TW871 fed a HFD for various periods of time.

**Methods**: First, 6-week-old C57BL/6J mice were fed an HFD for 4 weeks, then TW871 (1 × 10^7^ CFU) or phosphate-buffered saline (PBS) were intravenously administered. Mice were euthanized 12, 16, 20, and 48 weeks after starting the experiment, and conventional clinical and histopathological evaluations were performed.

**Results**: Typical NASH-like findings were not identified in the mice at 12 weeks, while they were observed in the TW871 group at 16 weeks, and the severity of NASH symptoms were increased at 20 weeks. Furthermore, signs of severe NASH were also observed at 48 weeks. In contrast, in the PBS-administered group, the NASH findings were identified only at 48 weeks and no typical NASH features were observed at 12, 16, or 20 weeks.

**Conclusion**: These results suggest that intravenous administration of a specific *S. mutans* strain aggravates NASH in a time-dependent manner in the mice in contrast to mice without *S. mutans* exposure.

## Introduction

*Streptococcus mutans*, a Gram-positive facultative anaerobic bacterium, is a pathogen associated with dental caries [1]. Moreover, the cell-surface protein antigens of *S. mutans*, including glucosyltransferases, 190-k Da protein antigens (PA), and glucan-binding proteins, are known to be associated with the severity of dental caries [2–4]. In addition, Cnm, a collagen-binding protein, has been purified and its encoding gene, *cnm*, sequenced [5]. Cnm is associated with the severity of infective endocarditis due to its modulation of the adhesive and invasive properties of the bacteria for blood vessel endothelial cells [6,7]. Furthermore, Cnm-positive *S. mutans* is associated with aggravation of cerebral haemorrhage and inflammatory bowel disease [8,9].

Steatohepatitis caused by lifestyle-related diseases, such as obesity, diabetes, and hyperlipidaemia, has received increasing attention [10]. Steatohepatitis is classified into alcoholic and non-alcoholic types, and non-alcoholic fatty liver disease (NAFLD) is further classified into simple steatosis and non-alcoholic steatohepatitis (NASH) [11,12]. NASH was first reported as a disease showing similar pathological findings to alcoholic steatohepatitis, leading to hepatic cirrhosis and cancer, with unknown aetiology [11,13]. The ‘two-hit theory’ is widely accepted as the mechanism of NASH development, in which insulin-resistance due to metabolic syndrome caused by excess nutrition leads to simple steatosis as the first step. Oxidative stress, lipid peroxidation, and functional failure of mitochondria accelerate inflammation and fibrosis in the liver as the second step, causing NASH [14].

It has been established that feeding 6-week-old C57BL/6J mice a high-fat diet (HFD) for 48 weeks results in a NASH-like condition [15]. We previously utilized several *S. mutans* strains with expression of Cnm and PA on the cell surface, which demonstrated that intravenous administration of a Cnm-positive PA-positive *S. mutans* strain to 6-week-old C57BL/6J mice induced NASH-like findings at 12 weeks and that the expressions of interferon-gamma considered to induce immune responses and metallothionein associated with oxidative stress were elevated in liver tissue [16,17]. Further studies showed that Cnm contributes to the adhesion of *S. mutans* to liver cells and PA enhances the affinity of liver cells for unsaturated fatty acids, resulting in their accumulation in the liver tissue [17]. Thus, administration of *S. mutans* with expression of Cnm and PA on the cell surface could play a role as the second hit of the ‘two-hit theory’ [17].

Previous studies demonstrated that Cnm-positive PA-positive *S. mutans* contribute to NASH development [16,17]. However, findings were limited to 16 weeks in the NASH mouse model. In the present study, evaluation of the mouse model before and after 16 weeks was undertaken to elucidate the detailed pathological process of *S. mutans*-associated NASH development.

## Materials and methods

### Bacterial strain and culture conditions

*S. mutans* TW871 (serotype *k*; Cnm (+), PA (+)), isolated from the blood of a patient with infective endocarditis complicated with subarachnoid haemorrhage [18], was used. The strain was cultured on mitis-salivarius-bacitracin (MSB) agar (Difco Laboratories, Detroit, MI, USA) plates containing bacitracin (0.2 U/ml; Sigma Chemical Co., St. Louis, MO, USA) and 15% (wt/vol) sucrose (MSB agar), or brain heart infusion (Difco) broth.

### Animal experiments

All mice were treated humanely in accordance with the National Institute of Health and AERI-BBRI Animal Care and Use Committee guidelines. All procedures used in the present study were approved by the Animal Care and Use Committee of the Osaka University Graduate School of Dentistry. The effects of intravenous administrations of *S. mutans* strains on the development of NASH were analysed using a NASH mouse model as previously described [15,19] with some modifications [17]. Briefly, 6-week-old C57BL/6J male mice (*n* = 94; Charles River Japan, Tokyo, Japan) were randomly divided into either the HFD plus phosphate-buffered saline (PBS) administration group (*n* = 42) or HFD plus TW871-treated group (*n* = 52). Mice were allowed free access to water and food throughout the experimental period. HFD 32 (Japan CLEA Inc., Tokyo, Japan), which contains 506.8 kcal/100 g (57.5% from fat, 19.7% from protein, and 22.8% from carbohydrate), was used to feed the mice in the HFD groups. The levels of the saturated and unsaturated fatty acids in the HFD groups were 22.3% and 76.9%, respectively. Four weeks after beginning the HFD, 1 × 10^7^ CFU of the *S. mutans* strain TW871 suspended in 100 µl of PBS or PBS without the added bacteria was intravenously injected via the jugular vein.

Eight, 12, 16, and 44 weeks after administration (12, 16, 20, and 48 weeks after beginning the HFD), mice were euthanized, and whole-body weights, liver weights, and visceral fat weights were measured (Supplementary Figure S1). Whole-body weight increase was calculated based on the whole-body weights at 0 weeks. In addition, serum levels of aspartate aminotransferase (AST) and alanine aminotransferase (ALT) were measured by FALCO Biosystems Ltd. (Kyoto, Japan). The results are expressed as the mean ± standard error of the mean (SEM). Tissue samples were fixed in 3.7% formaldehyde in PBS, embedded in paraffin, and cut into 3-µm sections for histopathological analysis. Haematoxylin–eosin (HE) staining was performed to evaluate the infiltration of inflammatory cells and adipocellular deposition, while Masson’s trichrome (MT) staining was used to evaluate fibrosis. Imaging and analyses of the sections were performed based on previously reported procedures [20], using microscope images obtained with 200× and 400× objectives (Olympus BX53, Olympus, Tokyo, Japan). Quantification of fat accumulation (Supplementary Figure S2) and fibrosis (Supplementary Figure S3) were performed by analysis of the histopathological images of HE- and MT-stained liver sections, respectively, using ImageJ software (version 1.43s; National Institutes of Health, Bethesda, MD, USA; available from http://rsbweb.nih.gov/ij/) as described previously [21]. The mean values of at least 10 randomly selected fields of view at 200× magnification were calculated. In addition, the numbers of Kupffer cells and liver cell nuclei were calculated in HE sections using 10 randomly selected fields of view at 400× magnification and the ImageJ software.

### Statistical analyses

Statistical analyses were performed using WinSTAT Statistics Software. All results are presented as the mean ± SEM. Intergroup differences for body weight, as well as the quantitative results of the histomorphometric analysis, were estimated using Bonferroni’s method following analysis of variance (ANOVA). *P*-values less than 0.05 were considered statistically significant.

## Results

### Aggravation of NASH in model mice induced by*S. mutans*strain TW871

**Figure 1.**
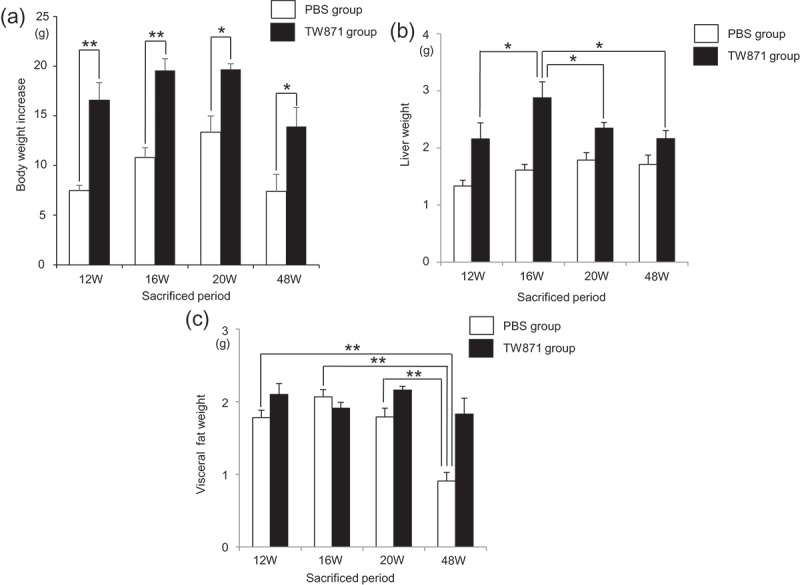
Effect of high-fat diet and *S. mutans* infection on mouse body weight increase (a), liver weight (b), and visceral fat weight (c). There was a statistically significant difference between two groups by Bonferroni’s method after ANOVA; *P < 0.05, **P < 0.01.

Macroscopic observations showed that the mice in the TW871 group were larger than those in the PBS group for each experimental period. Similarly, the mean whole-body weight of mice in the TW871 group was significantly greater than that of the PBS group at each time point (*P *< 0.05) (Figure 1(a)). In fact, no notable weight gain was observed after 16 and 20 weeks in both groups, and body weight was decreased at 48 weeks to levels similar to those at 12 weeks. The mean liver weight of the TW871 group plateaued at 16 weeks, which was significantly greater than that at 12, 20, and 48 weeks (*P* < 0.05) (Figure 1(b)). The mean visceral fat weights of the PBS group at 12, 16, and 20 weeks were similar, which was significantly reduced at 48 weeks (*P* < 0.01) (Figure 1(c)).

**Figure 2.**
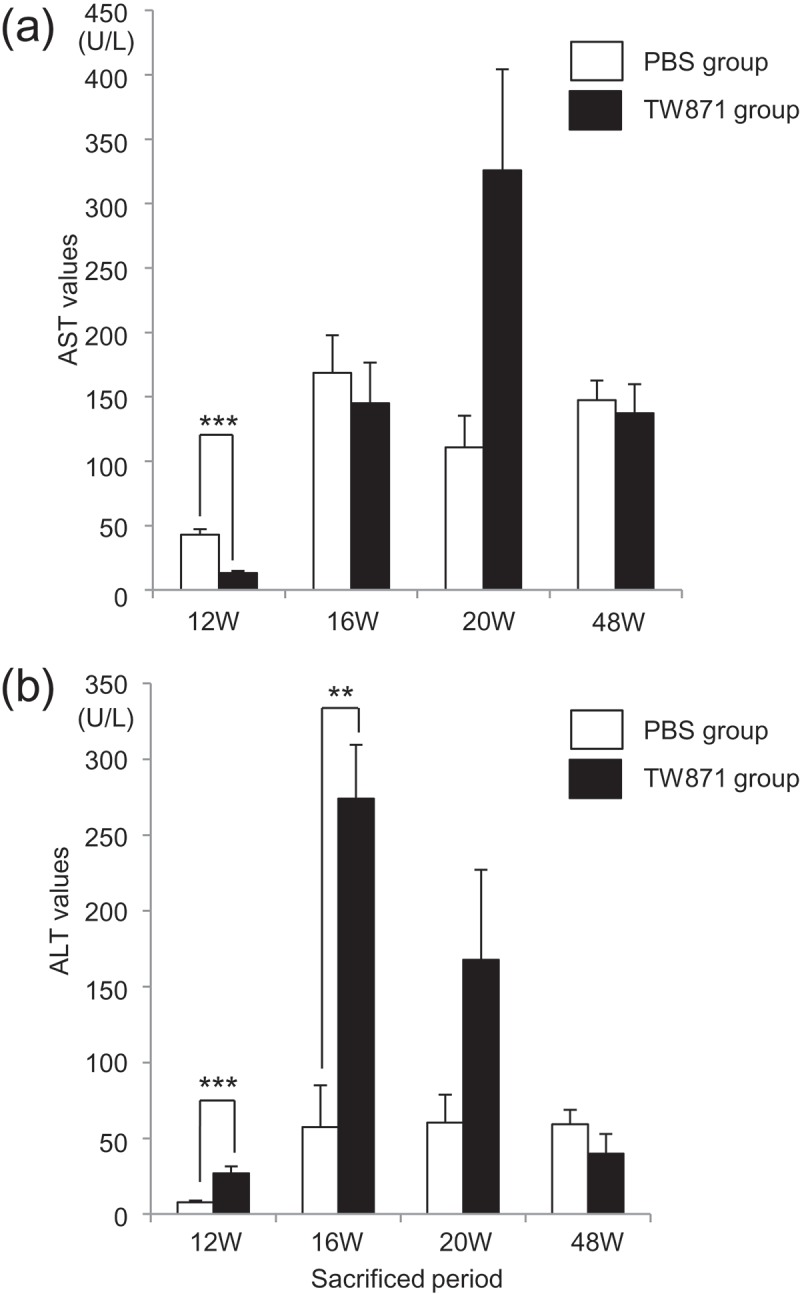
Serum AST and ALT levels in mice fed a high-fat diet and mice administered *S. mutans*. (a) AST. (b) ALT. There was a statistically significant difference between two groups by Bonferroni’s method after ANOVA; **P < 0.01, ***P < 0.001.

As for markers of liver damage, the AST levels were significantly increased in the PBS group at 12 weeks compared with the TW871 group (*P *< 0.001). However, there were no significant differences in AST between the two groups at 16, 20, and 48 weeks (Figure 2(a)). In contrast, the ALT values of the TW871 group were significantly greater at 12 and 16 weeks compared with the PBS group (*P *< 0.01), although there was no significant difference between the two groups at 20 and 48 weeks (Figure 2(b)).

### Histopathological findings in the NASH mouse model

**Figure 3.**
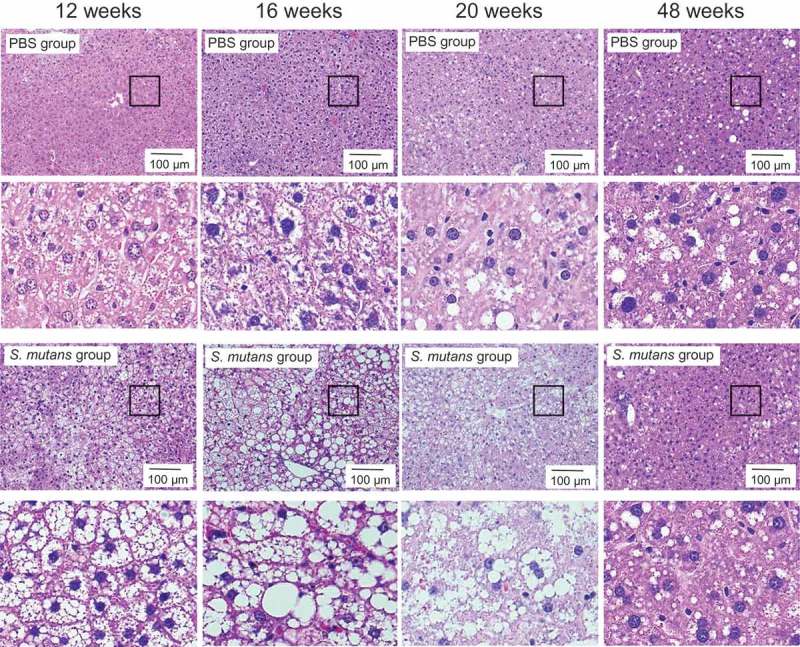
Representative histopathological findings in liver tissue after haematoxylin–eosin staining in mice fed a high-fat diet and administered *S. mutans*. Lower panels are magnification of the rectangular area of each upper panel.

**Figure 4.**
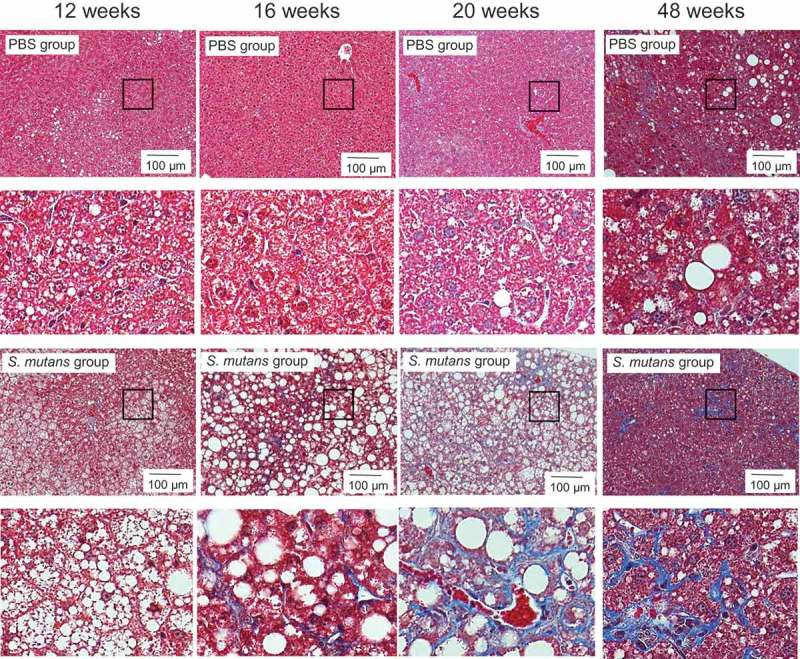
Representative histopathological findings in liver tissue after Masson’s trichrome staining in mice fed a high-fat diet and mice administered *S. mutans*. Lower panels are magnification of the rectangular area of each upper panel.

**Figure 5.**
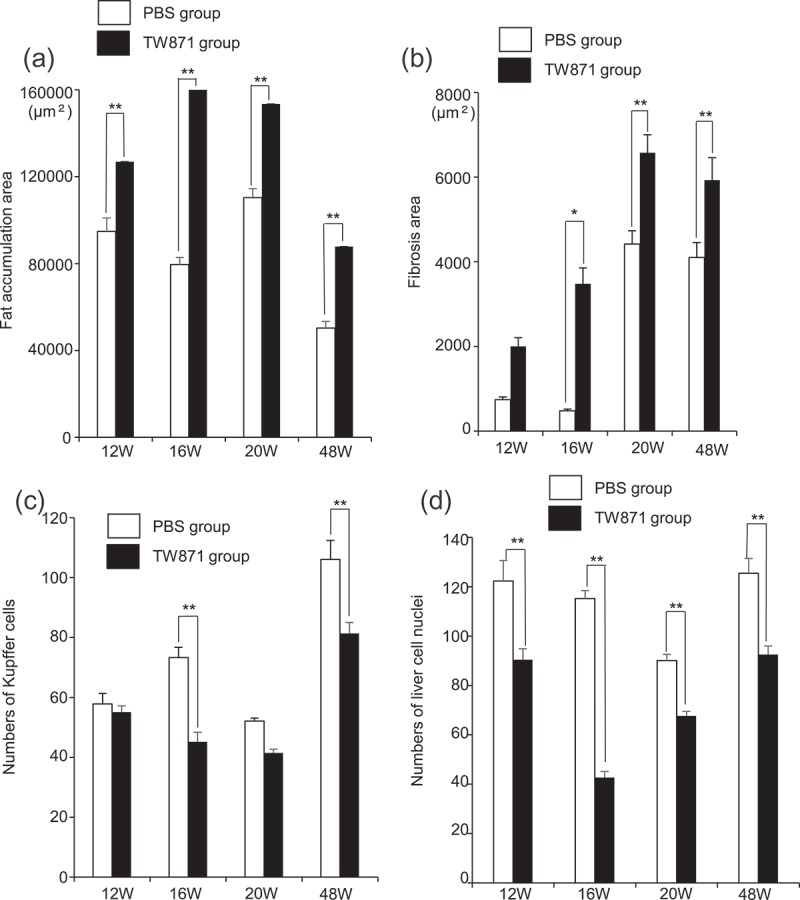
Quantification of histopathological images using ImageJ in mice fed a high-fat diet and mice administered *S. mutans*. (a) Fat accumulation. (b) Fibrosis. (c) Kupffer cells. (d) Liver cell nuclei. There was a statistically significant difference between two groups by Bonferroni’s method after ANOVA; *P < 0.05, **P < 0.01.

HE staining of liver sections demonstrated that fat accumulation was observed more widely in the TW871 group than in the PBS group at each time point (Figure 3). In the MT-stained sections, prominent fibrosis was observed in perivascular and liver parenchyma at 16, 20, and 48 weeks in the TW871 group, which was not observed in the PBS group (Figure 4). After quantifying the histopathological images using ImageJ, the area of fat accumulation in the liver tissue in the TW871 group was significantly greater than that of the PBS group at each time point (*P *< 0.01) (Figure 5(a)). In addition, the TW871 group showed a significantly greater area of fat accumulation at 16 and 20 weeks as compared with 12 weeks, whereas it was decreased at 48 weeks. Similarly, the area of fibrosis in the TW871 group was significantly greater than that of the PBS group at 16, 20, and 48 weeks (*P *< 0.05) (Figure 5(b)). In both groups, fibrosis reached a peak at 20 weeks. The number of Kupffer cells in the livers of the TW871 group was significantly lower than that of the PBS group at 16 and 48 weeks (*P* < 0.01) (Figure 5(c)). Furthermore, the number of liver cell nuclei in the TW871 group at each time point was significantly lower than that of the PBS group (*P *< 0.01) (Figure 5(d)). Notably, the number of liver cells in the TW871 group was lowest at 16 weeks.

## Discussion

This is the first study in which the kinetics of *S. mutans*-associated NASH aggravation have been evaluated. It had been reported previously that 48 weeks are needed to observe NASH-like findings in the HFD mouse model [15]. In contrast, NASH-like findings were obtained only 16 weeks after the administration of *S. mutans* strain TW871 in mice fed an HFD. The present study evaluated the mice before and after 16 weeks of an HFD. In addition, quantification of important histopathological findings of NASH, such as fat accumulation and fibrosis in the liver tissues, was performed by image analysis. Furthermore, the numbers of Kupffer cells and hepatic cell nuclei, markers of liver disease [22,23], were calculated. These approaches enable a better understanding of the details of *S. mutans*-associated NASH development.

NASH-like features were prominently observed at 16 weeks in the TW871 group, which became more prominent as time progressed. In the PBS group, NASH-like features were only observed at 48 weeks, with no findings related to NASH in weeks 12, 16, and 20, confirming a previous report [15]. Since the present study demonstrated that administration of TW871 accelerated NASH as compared with PBS, it is reasonable to expect that the body weights of the TW871 group were significantly greater than that of the PBS group at various time points. However, the mean body weight of the PBS group peaked at 20 weeks and was reduced at 48 weeks, whereas the TW871 group plateaued at 16 weeks but was also reduced at 48 weeks. These changes are hypothesized to be due to the worsening systemic conditions at 48 weeks caused by NASH development in both groups. Especially, mean liver weight of the TW871 group plateaued at 16 weeks and was reduced at 20 and 48 weeks, which was estimated to be derived from the development of fibrosis as the time goes by. In addition, visceral fat weight at 48 weeks in the PBS group was reduced as compared with 12, 16, and 20 weeks, leading to the reduced body weight increase at 48 weeks.

As for ALT concentration, a marker for evaluation of initial NASH development [24,25], the values of the TW871 group were significantly greater than those of the PBS group at 12 and 16 weeks. However, ALT values at 20 and 48 weeks were decreased as compared with 16 weeks in the TW871 group, and were similar to those observed in the PBS group over the entire period. These results are consistent with a previous report, in which the ALT values in severe NASH returned to the level of healthy subjects [26].

Histopathological evaluation of the liver tissues revealed prominent fat accumulation in the TW871 group over the entire period and the initiation of fibrosis at 16 weeks which was aggravated as time progressed. In contrast, there was no fat accumulation and fibrosis at 12, 16, and 20 weeks, and only slight fat accumulation and fibrosis observed at 48 weeks in the PBS group. Quantification by image analysis enabled a clearer understanding of the disease at all time points in the present study. These histological results are also supported by the body weight and serum ALT and AST results. The numbers of Kupffer cells and hepatic cell nuclei were evaluated in the present study since they could be calculated by the image processing software. Kupffer cells are widely distributed in liver tissues, and are also associated with the initiation of NASH [22]. In fact, the number of the Kupffer cells was reduced in the TW871 group at 16 and 48 weeks. Although there are no reports regarding the reduction of cell number in hepatic tissues, it is hypothesized that hepatocyte numbers decrease due to atrophy and necrosis in the final stages of NASH. The present study demonstrated that the hepatocyte numbers in the TW871 group were significantly lower than in the PBS group, with the lowest values at 16 weeks when NASH-like findings were initially identified. This result suggests that vacuolation of the nucleus caused by the accumulation of fat in the liver cells and acceleration of fibrosis could induce the reduction in cell numbers.

A limitation of the present study is that the mouse model was only evaluated after a single administration of *S. mutans* strain TW871. It is known that invasive dental treatments, such as tooth extraction and periodontal surgery, cause bacteraemia during the procedure [27]. In that report, it was estimated that more than 1 × 10^4^ CFU/mL bacteria are present in the bloodstream in approximately 60% of human subjects after invasive dental treatment, which is equivalent to more than 5 × 10^7^ CFU in the entire body. In addition, random bacteraemia occurs after tooth brushing and flossing during daily life [28]. This information led us to consider that multiple administration of the bacteria should be evaluated in further studies. We previously confirmed that less than 1 × 10^6^ CFU of *S. mutans* TW871 could not cause aggravation of NASH in this mouse model [17]. In contrast, it was reported that 1 mg of dental plaque contains more than 1 × 10^7^ CFU bacteria [29], suggesting the 1 × 10^7^ CFU bacteria used in the present study could enter the bloodstream when bacteraemia occurs.

Unfortunately, we have no data regarding the change of the microbiological environment after administration of TW871. We emphasize that not only a specific strain of *S. mutans* but also other species could possibly induce aggravation of NASH. In fact, a specific strain of *Porphyromonas gingivalis*, one of the major periodontitis-related species, also aggravates NASH conditions in the same mouse model [19]. We will investigate the effects of other bacterial species to the development of NASH conditions in future studies. In addition, we have no data regarding the inflammatory status of the whole body in the TW871 group. We will therefore evaluate the inflammatory as well as related markers regarding NASH by blood serum biochemical examinations in future studies. Furthermore, when discussing intravenous administration of a specific type of *S. mutans* as a factor of the second hit in the two-hit theory, we could have examined pro-inflammatory cytokine levels in each time point. This could be the limitations when interpreting the results of the present study.

In summary, the present results demonstrate that a single administration of a specific *S. mutans* strain at 4 weeks triggers the initiation of NASH as the second hit in the ‘two-hit theory’ [14], causing NASH-like features at 16 weeks and leading to fibrosis by 20 weeks, which became severe by 48 weeks. In contrast, an HFD alone only caused NASH-like findings after 48 weeks. Therefore, invasion of the bloodstream by *S. mutans* could be a possible second hit in the ‘two-hit theory’, which accelerates NASH development from simple steatosis. Further studies will focus on the distribution of this specific type of *S. mutans* in NASH patients.

## Supplementary Material

Supplementary_data.zip
