# Supplementary material for: Longitudinal comparison of Streptococcus mutans-induced aggravation of non-alcoholic steatohepatitis in mice
Source: J Oral Microbiol. 2018 Jan 22;10(1):1428005. doi: 10.1080/20002297.2018.1428005 (PMC5795759; doi:10.1080/20002297.2018.1428005)
Supplement: Supplementary_data.zip [file ZJOM_A_1428005_SM4407.zip › Supplementary data/Supplementary Figure1-rev.pptx]

## Slide 1
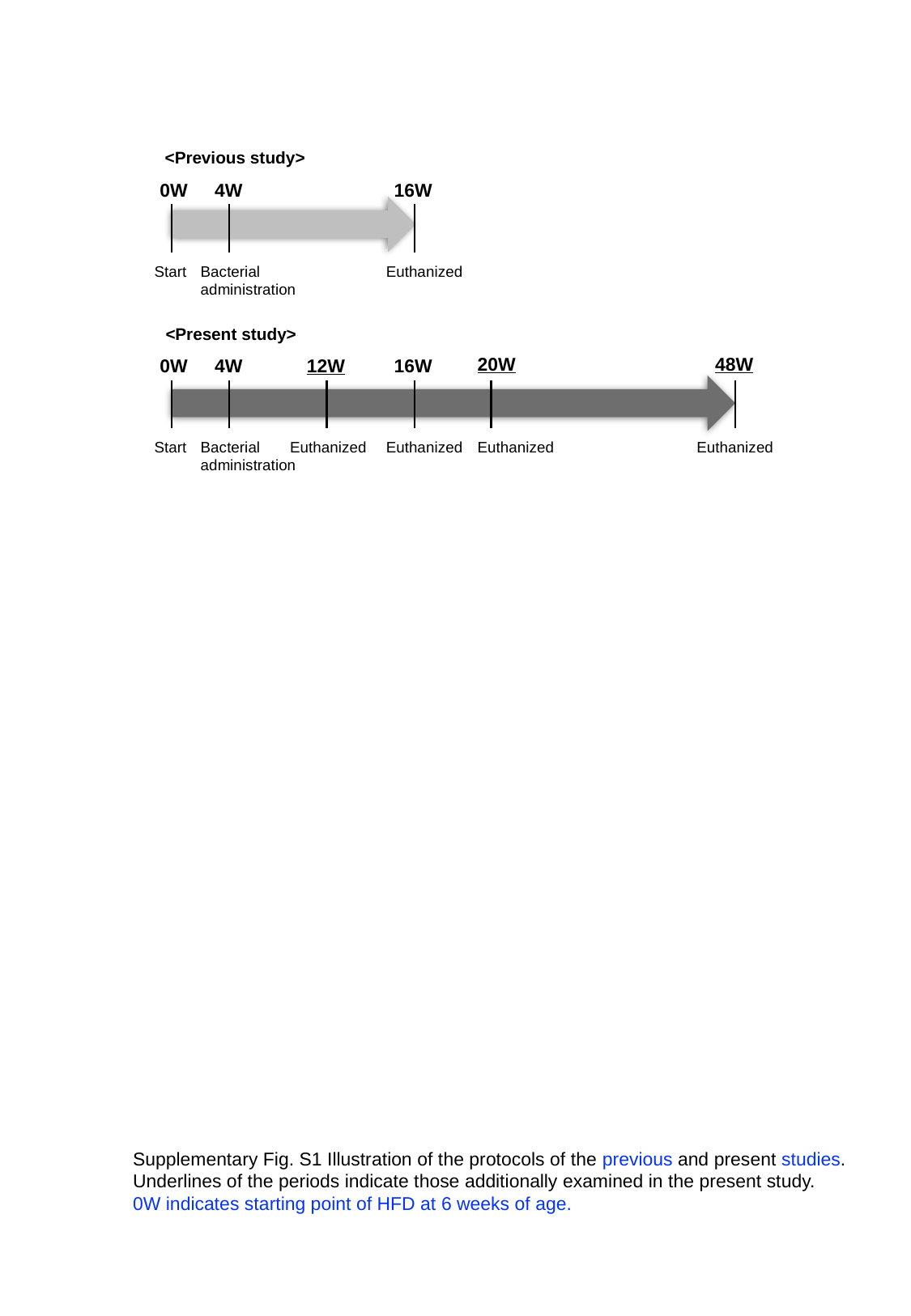

<Previous study>
0W
4W
16W
Start
Bacterial
administration
Euthanized
<Present study>
20W
48W
0W
4W
12W
16W
Start
Bacterial
administration
Euthanized
Euthanized
Euthanized
Euthanized
Supplementary Fig. S1 Illustration of the protocols of the previous and present studies.
Underlines of the periods indicate those additionally examined in the present study.
0W indicates starting point of HFD at 6 weeks of age.
