# Supplementary material for: Longitudinal comparison of Streptococcus mutans-induced aggravation of non-alcoholic steatohepatitis in mice
Source: J Oral Microbiol. 2018 Jan 22;10(1):1428005. doi: 10.1080/20002297.2018.1428005 (PMC5795759; doi:10.1080/20002297.2018.1428005)
Supplement: Supplementary_data.zip [file ZJOM_A_1428005_SM4407.zip › Supplementary data/Supplementary Figure2-rev.pptx]

## Slide 1
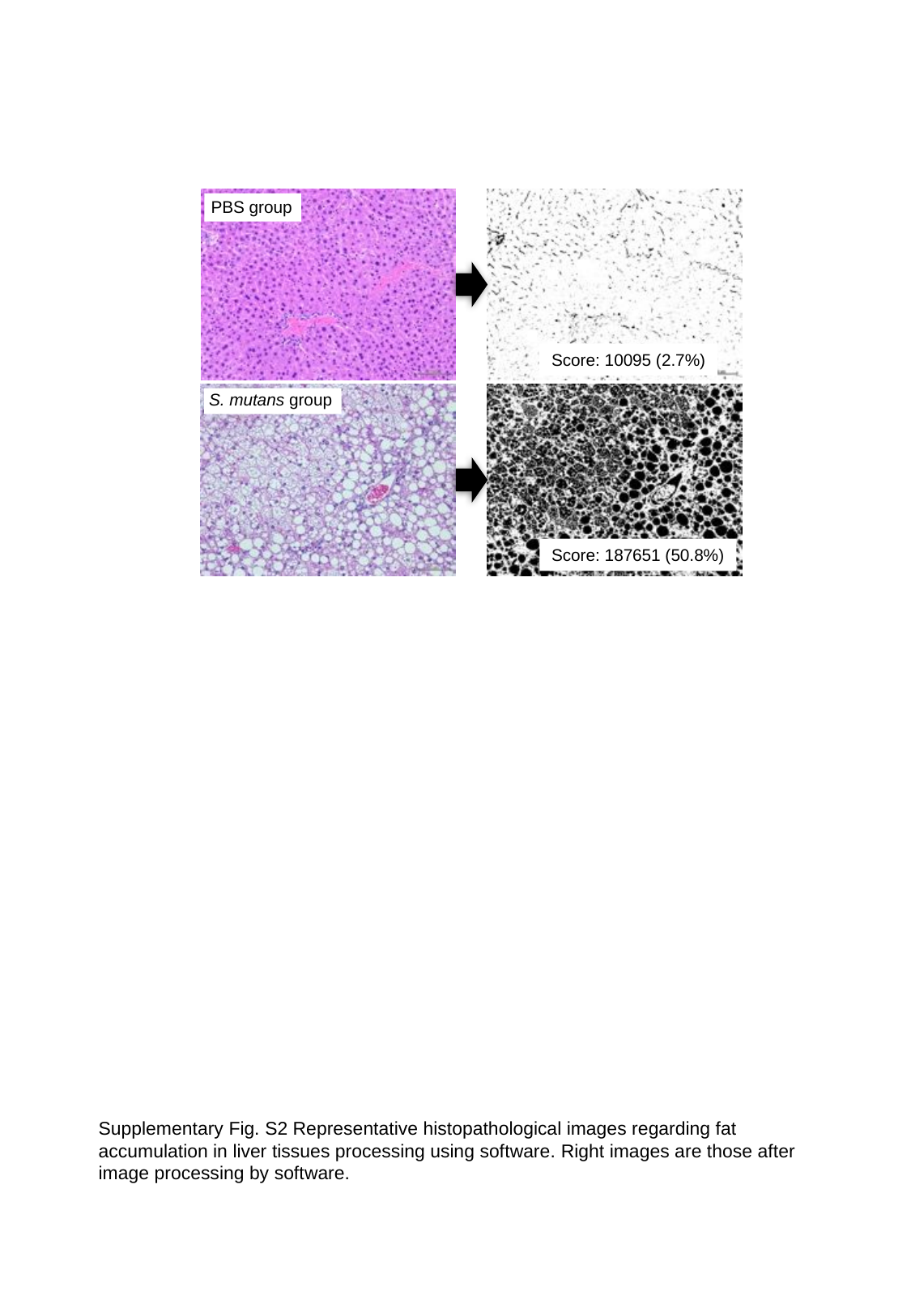

PBS group
Score: 10095 (2.7%)
S. mutans group
Score: 187651 (50.8%)
Supplementary Fig. S2 Representative histopathological images regarding fat accumulation in liver tissues processing using software. Right images are those after image processing by software.
