# Supplementary material for: Longitudinal comparison of Streptococcus mutans-induced aggravation of non-alcoholic steatohepatitis in mice
Source: J Oral Microbiol. 2018 Jan 22;10(1):1428005. doi: 10.1080/20002297.2018.1428005 (PMC5795759; doi:10.1080/20002297.2018.1428005)
Supplement: Supplementary_data.zip [file ZJOM_A_1428005_SM4407.zip › Supplementary data/Supplementary Figure3-rev.pptx]

## Slide 1
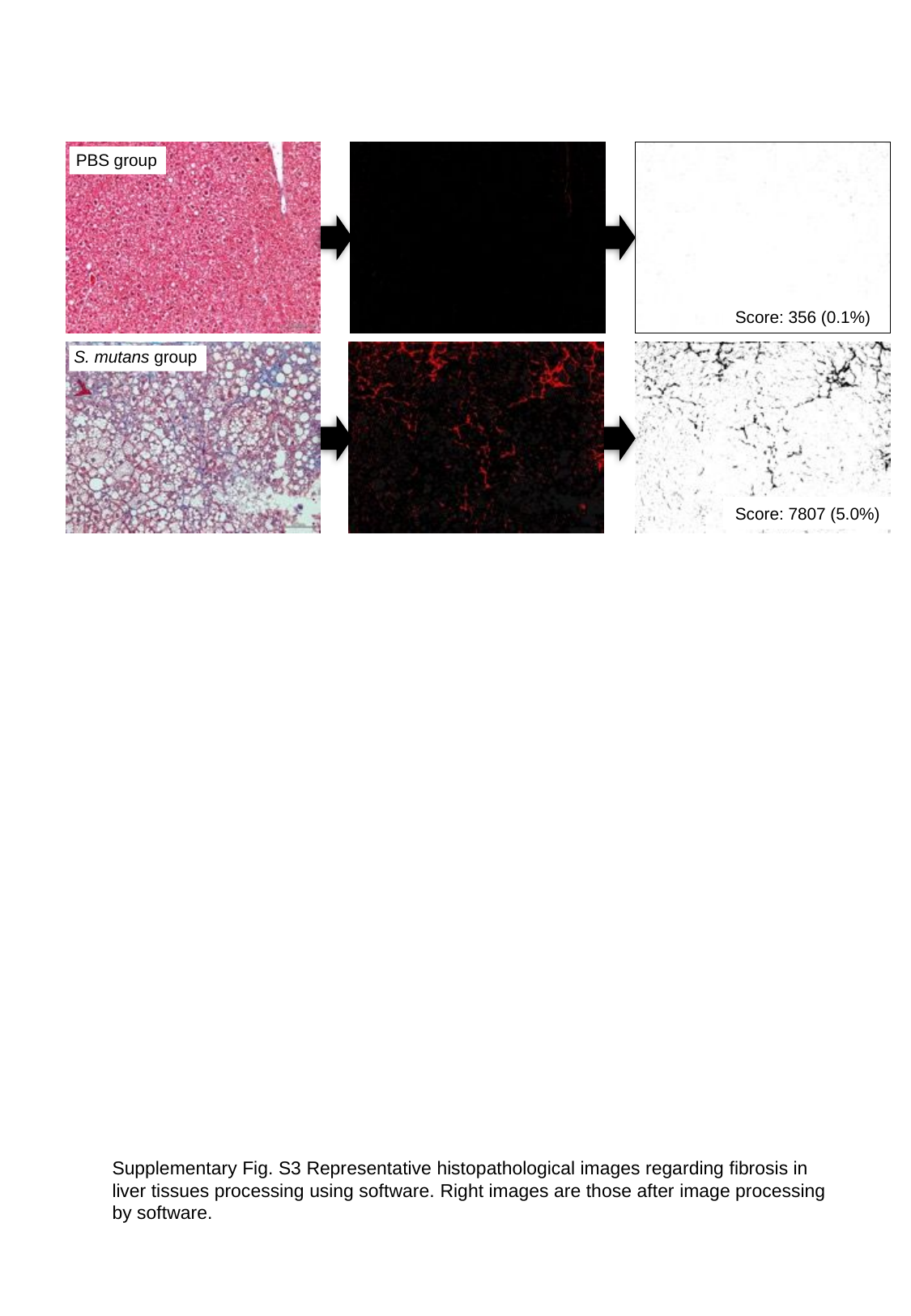

PBS group
Score: 356 (0.1%)
S. mutans group
Score: 7807 (5.0%)
Supplementary Fig. S3 Representative histopathological images regarding fibrosis in liver tissues processing using software. Right images are those after image processing by software.
